# Ankle-Specific Training Does Not Alter Drop Jumping Biomechanics Despite Increased Plantar Flexor Strength and Jumping Performance

**DOI:** 10.7759/cureus.42228

**Published:** 2023-07-20

**Authors:** Theodoros M Kannas, Eirini Argiriadou, Georgios Chalatzoglidis

**Affiliations:** 1 Laboratory of Neuromechanics, Department of Physical Education and Sport Science, Aristotle University of Thessaloniki, Thessaloniki, GRC; 2 Department of Physical Education and Sport Science, Aristotle University of Thessaloniki, Thessaloniki, GRC

**Keywords:** isokinetic torque, contact time, vertical jump, plantar flexors, vertical jumping, plane hopping, incline hopping, electromyostimulation, plyometric training

## Abstract

Introduction: Power plays a crucial role in determining an athlete's final performance, as it signifies the ability to rapidly generate force. The plantar flexor muscles have a crucial role in producing the necessary power. The plantar flexor muscles are important in explosive sports movements due to their ability to generate substantial force quickly during the propulsion phase and facilitate efficient energy transfer through the joints. This study aimed to investigate the effects of specific plantar flexor training on drop jumping (DJ) biomechanics, muscle activation, and muscle strength.

Material and methods: A total of 30 male participants were divided into three groups: the incline hopping (IH) group, which performed continuous jumps on a 15° inclined surface; the plane hopping (PH) group, which performed jumps on a plane surface; and the electrostimulation (EMS) group (n = 10 for each group). All groups trained four times weekly, performing 10 sets of 10 jumps per session. The intervention period lasted four weeks. Participants' drop jumping ability was assessed before and immediately after the training period using hip, knee, and ankle kinematics and electromyographic (EMG) activity of the medial gastrocnemius (MGas), tibialis anterior (TA), rectus femoris (RF), and semitendinosus (ST) muscles. In addition, maximal isokinetic plantar flexor force measurements were evaluated in eccentric and concentric conditions.

Results: Analysis of variance (ANOVA) revealed that only the inclined hopping showed significant improvements in the take-off velocity (V_to_) of the fast drop jump (bounce drop jump (BDJ)) (p < 0.05). These improvements were accompanied by significantly higher MGas activity during the propulsion phase of the jump (p < 0.05). In addition, all groups demonstrated greater eccentric torque (p < 0.05), while IH also improved concentric torque (p < 0.05).

Conclusions: The results support the efficacy of inclined hopping in improving the V_to_ of BDJs. The increased MGas activity and stable co-activation index (CI) during the propulsion phase are likely to contribute to these improvements. Coaches should consider incorporating incline hopping into the periodization of athletes, while level hopping and electrostimulation could be used to increase overall strength.

## Introduction

Power plays a crucial role in determining an athlete's final performance, as it signifies the ability to rapidly generate force [1]. For athletic actions such as sprinting, jumping, and throwing, where there is a need for high force production in a short contact time (CT), having a high power capacity is essential [2]. While various muscle groups contribute to these movements, the plantar flexor muscles have a critical role in supplying the necessary power output. The plantar flexor muscles are particularly important in explosive sports movements due to their ability to generate substantial force quickly during the propulsion phase and facilitate efficient energy transfer through the joints. The Achilles tendon acts as an amplifier, amplifying power through a catapult-like action under optimal conditions [3]. Consequently, optimizing explosive sports performance relies on the power production by the plantar flexors and its transmission through the Achilles tendon.

Power training encompasses exercises such as plyometrics, Olympic lifts, and explosive drills, which aim to enhance the rate of force development, power production, and overall performance [4]. Plyometric training is a fundamental component of sports conditioning, involving dynamic movements and substantial power production [5]. It utilizes forceful muscular contractions in a short duration, employing the stretch-shortening cycle to generate maximal power [6]. Plyometric training has been shown to alter the level of activation in the pre-activation phase, eccentric phase, and concentric phase of jumping [7-9]. Moreover, it was previously established that it promotes neural adaptations that improve the efficiency and coordination of muscle contractions during counter movement jump (CMJ), but not in drop jump (DJ) [10]. Additively, a previous study indicated that plantar flexor's activation increased after both plyometric and weight training while antagonist muscles remain unaffected [10]. However, the available data on the effects of plantar flexors' activation alterations on joint biomechanics during jumping is currently insufficient.

Hopping is commonly employed in plyometric training to enhance power production in the plantar flexors, despite that its exclusive use does not provide sufficient stimulus to change the jumping performance [9]. Conversely, inclined hopping (IH) has been shown to induce earlier adaptations in fast drop jumping by optimizing the force-length relationship and force-velocity relationship of the plantar flexors [11]. Furthermore, another study demonstrated that plane hopping (PH) and electromyostimulation (EMS) training led to altered muscle co-activation during slow stretch-shortening cycle jumping, whereas inclined plyometric training did not affect muscle co-activation [12]. Moreover, a previous study showed that a six-week plyometric intervention (with 720 jumps in total) led to a higher extension of the hip and the knee accompanied by lower hip angular and higher knee extension velocity [13]. Similarly, Olympic lifts were found to increase the maximum flexion of the hip and knee, while strength training causes a decrease [7]. Therefore, inclined hopping should be considered a specific exercise targeting the plantar flexors. However, the effects of hopping on the interaction between knee and ankle biomechanics have not been adequately studied.

In recent decades, local or whole-body EMS training has been systematically used for athletic performance development [14] and rehabilitation progress [15]. Applying EMS under isometric conditions to specific muscle groups appears to be a promising method for improving athletic capacity and jumping performance. While previous studies have demonstrated increased muscle activation and force production through isometric target EMS on specific muscle groups such as the quadriceps and calf muscles [16], the performance of multi-joint explosive actions remains unaffected [17]. EMS training, in conjunction with plyometric training, exhibited significant enhancements in jumping performance, as demonstrated by previous findings [18]. A previous study [19] also yielded similar outcomes, illustrating that when combined with rugby training, EMS training resulted in enhanced strength and jumping capacity, with no discernible alterations in sprinting actions. However, despite the established benefits of EMS training on vertical jumps, the existing body of knowledge lacks information regarding its impact on jumping biomechanics. Consequently, this study aimed to ascertain the effects of various specialized plantar flexor training regimens on the biomechanics of vertical jumping.

## Materials and methods

Participants

The power analysis was conducted using G*Power software (v.3.1.9.4) to determine the required sample size for repeated measures analysis of variance (ANOVA). The analysis aimed to examine the effects of three types of training (IH, PH, and EMS) on the dependent variable of drop jump. The input parameters included an effect size estimated from pilot data (partial eta-squared = 0.35), an alpha level of 0.05, and a power level of 0.80. The analysis revealed that a minimum sample size of 24 (eight participants/group) participants would be needed to achieve the desired power. The assumption of a correlation of 0.5 between pre- and post-measurements within each group was made. This sample size estimation was based on the assumption that the effect size observed in the pilot study would generalize to the larger sample. Male students in the age group of 17-20 years with no systematic training in a specific sport for the last two years, who were in active state (10-12 hours/week) participating only in the department's courses (same semester), with no systematic strength or plyometric training in the last six months, and were willing to participate, were included in the study. In contrast, smokers and students using food supplements such as protein or creatine, or having a lower limb injury in the last six months were excluded from the study. Finally, 30 male students of the Department of Physical Education and Sport Science at Serres participated in this study. Approval for the experiment was obtained from University Ethics Committee on Human Research (Local Ethics Research Committee (ERC- 008/2020)), and the participants were informed about the experimental procedure and signed their written consent prior to participation.

Procedure

Participants were randomly (Random Generator, free software) assigned to the IH group (n = 10), the PH group, and the EMS group (n = 10). A total of 16 sessions (four sessions/week, four weeks) were performed by the experimental groups. The IH and PH performed one session, to be familiarized with the jumping technique. Each plyometric training session was divided into three phases. The warm-up phase included jogging and stretching exercises. The main phase included 10 sets of 10 maximum jumps, with a two-minute rest. During the main phase, the IH group performed hopping on an inclined (15°) ramp. The PH group performed hopping on a plane ramp. Special attention was given to executing the jumps "as fast and high as possible." The recovery phase included five-minute stretching. The EMS group also participated in one session to be familiar with stimulation. They completed 16 sessions (30 minutes) of isometric bilateral EMS (Myostim, Medicompex, Ecublens, Switzerland) over the training period. Eighty isometric contractions were performed during each training session. The participants were standing against the wall with the ankle at -15° (dorsiflexion) and the knee fully extended while the hands touched the wall. Each session was preceded by a standardized warm-up consisting of five minutes of submaximal EMS (5 Hz; pulses lasting 200 msec). Two positive electrodes (5 × 5 cm) were placed over the superficial aspects of the medial gastrocnemius (MGas) and lateral gastrocnemius (LGas), while negative electrodes (10 × 5 cm) were placed over their muscles' bellies. Each four-second contraction was followed by a pause lasting 20 seconds. Intensity (range: 0-100 mA) was monitored online and gradually increased to maximally tolerated intensity depending on the subjects' threshold. The whole intervention process has been described in detail in a previous study [12].

Data collection and analysis

After warm-up (10-minute jogging, static stretching, and various submaximal vertical jumps and jumping drills), participants were asked to perform three maximum DJs from 20, 40, and 60 cm in a randomized order. Since the degree of knee flexion during landing determines the contribution of the different muscle groups (knee extensors versus plantar flexors) [9], all DJs were performed under two different task conditions defined by the following instructions: counter drop jumps (CDJ) for the slow, "jump as high as possible after landing," and bounce drop jumps (BDJ) for the fast, "jump as fast as possible." A twin-axis electronic goniometer (Biopac Systems Inc., Goleta, CA, USA) was used to record knee joint angles during all jumping tests. During the test, participants were instructed to minimize upper body movement. DJs were considered as being "slow" or "fast" when the maximum knee joint angle was greater than 60° or less than 50°, respectively. The best jump based on take-off velocity (V_to_) was used for further analysis. All tests were supervised by the same investigator, and standardized verbal encouragement was provided to all participants ("as high as" and "as fast and high" for CDJ and BDJ, respectively). Contact time (CT) was also taken as a measure of jumping performance.

Kinematics

Kinematic data were collected using the 3D Vicon motion analysis system (Oxford Metrics Ltd., Oxford, UK). The camera was calibrated to a volume of 2.0 m^3^, and calibration errors were all below 3 mm. Kinematic data were sampled at 120 Hz. Retroreflective spherical markers were placed on selected anatomical landmarks: the head of the fifth metatarsal, lateral malleolus, lateral femoral epicondyle, greater trochanter, and acromion. A standing trial was recorded to establish initial joint angle conditions. The resulting displacement-time data of each marker were filtered using a low-pass, two-order, Butterworth, dual-pass filter. The cutoff frequency for filtering the kinematic data was set at 8 Hz. For reference, full hip extension and knee extension were set equal to 180°, whereas the ankle in neutral position was equal to 0° (- indicated dorsiflexion and angles < 0° indicated plantarflexion). The V_to_ was calculated from the values of its vertical components.

Electromyography (EMG)

Four bipolar silver chloride surface electrodes (IOMED, Inc., Salt Lake City, UT, USA) (voltage range: +4 to +12 V) with 2 cm interface distance were placed on the muscle bellies of MGas, tibialis anterior (TA), rectus femoris (RF), and semitendinosus (ST) during jumping tests. The electromyographic (EMG) signals were amplified with a bandwidth frequency ranging from 1.5 to 2 kHz (common-mode rejection ratio = 90 IB, Z input = 1 LX, Gain = 1,000), filtered (cutoff: 10-500 Hz) and digitally sampled at a frequency of 1,000 Hz (full-wave rectification, fourth-order, zero lag Butterworth filter). The mean EMG (EMGmean) of the jumps was calculated by full-wave rectification and averaged over eccentric and concentric phases. For DJs, the movement was divided into eccentric and concentric phases using the changes in the angular position of the knee due to the kinematics. The mean value of MGas and RF EMG over the eccentric and concentric phase of the jumps was normalized with the EMGmean of the total MGas and RF activity, respectively. Antagonist co-activation was expressed as the ratio of maximum EMG activity of TA and ST to the maximum EMG value of MGas and RF, respectively, during the same phase of the jump [10] and referred to as the co-activation index (CI).

Isokinetic torque

After a standardized warm-up period, participants performed three maximal plantarflexions, with their dominant limb, in three concentric (30, 60, and 120°∙s-1) and three eccentric (30, 60, and 120°∙s-1) velocities randomly presented in an isokinetic dynamometer (Cybex 6000, Lynex Corporation, Ronkonkoma, NY, USA). Special attention was given to the stabilization of the participants, isolating the muscle group tested and properly aligning the limb and machine axes of rotation. All participants were positioned and secured according to the Cybex Multi-Joint System Manual. Consistent and identical verbal encouragement was provided during the test. A two-minute rest was allowed between trials to eliminate the effects of fatigue. The best of the three trials based on maximum produced torque was selected for further analysis. Participants were familiarized with the apparatus and testing procedure over a period of one week (three sessions of 40 minutes). All tests, before and after training, were performed at the same time of the day to avoid any chronobiological effect.

Statistical analysis

An analysis of variance (ANOVA) with repeated measurements (RM) on time (pre and post) was conducted using Statistical Package for the Social Sciences (SPSS) (v.28.0, IBM SPSS Statistics, Armonk, NY, USA) to examine the relationships between the effect of the three types of training "group" (three levels: IH, PH, and EMS) and depended value of drop jump. Significant time x group interaction was further analyzed using pre-post training differences (Tukey test). The significance level for all tests was set at p < 0.05.

## Results

The analysis (RM-ANOVA) revealed a non-statistically significant interaction between group and time for the dependent variables of CDJ (Table 1), while it revealed a statistically significant interaction between group and time for the dependent variables of BDJ (Table 1).

**Table 1:** Take-off velocity (m/s) values (mean ± SD) before and after the four-week intervention period for the three training groups * indicates significant changes (p < 0.05) compared with the pre-training values. V_to_: take-off velocity, CDJ20: counter drop jump from 20 cm, CDJ40: counter drop jump from 40 cm, CDJ60: counter drop jump from 60 cm, BDJ20: bounce drop jump from 20 cm, BDJ40: bounce drop jump from 40 cm, BDJ60: bounce drop jump from 60 cm, IH: incline hopping, PH: plane hopping, EMS: electromyostimulation, SD: standard deviation

	IH		PH		EMS	
V_to_ (m/s)	Before	After	Before	After	Before	After
CDJ20	2.53 ± 0.39	2.72 ± 0.11	2.57 ± 0.33	2.74 ± 0.10	2.53 ± 0.46	2.69 ± 0.76
CDJ40	2.66 ± 0.25	2.80 ± 0.13	2.59 ± 0.34	2.73 ± 0.14	2.63 ± 0.43	2.79 ± 0.48
CDJ60	2.55 ± 0.44	2.75 ± 0.11	2.47 ± 0.34	2.69 ± 0.50	2.57 ± 0.54	2.71 ± 0.93
BDJ20	2.76 ± 0.24	3.05 ± 0.77*	2.82 ± 0.26	2.93 ± 0.26	2.87 ± 0.53	2.99 ± 0.30
BDJ40	2.75 ± 0.24	3.02 ± 0.24*	2.75 ± 0.20	2.90 ± 0.26	2.81 ± 0.42	2.98 ± 0.35
BDJ60	2.71 ± 0.34	3.09 ± 0.39*	2.79 ± 0.19	2.92 ± 0.64	2.74 ± 0.36	2.89 ± 0.37

The V_to_ during BDJ20 ranged from 2.34 to 3.62 m/s. The ANOVA results indicated a significant two-way interaction effect (F_(2,27) _= 4,321, p < 0.05). The V_to_ during BDJ40 ranged from 2.51 to 3.24 m/s (Table 1). The ANOVA results indicated a significant two-way interaction effect (F_(2,27)_ = 4,149, p < 0.05). The V_to_ during BDJ60 ranged from 2.37 to 3.48 m/s. The ANOVA results indicated a significant two-way interaction effect (F_(2,27)_ = 4,560, p < 0.05). Post hoc analysis was conducted to further examine the specific effects within each group and time factor. The results indicated that V_to_ was significantly increased only in the IH group, while no significant effect was found for the PH and EMS groups.

**Figure 1:**
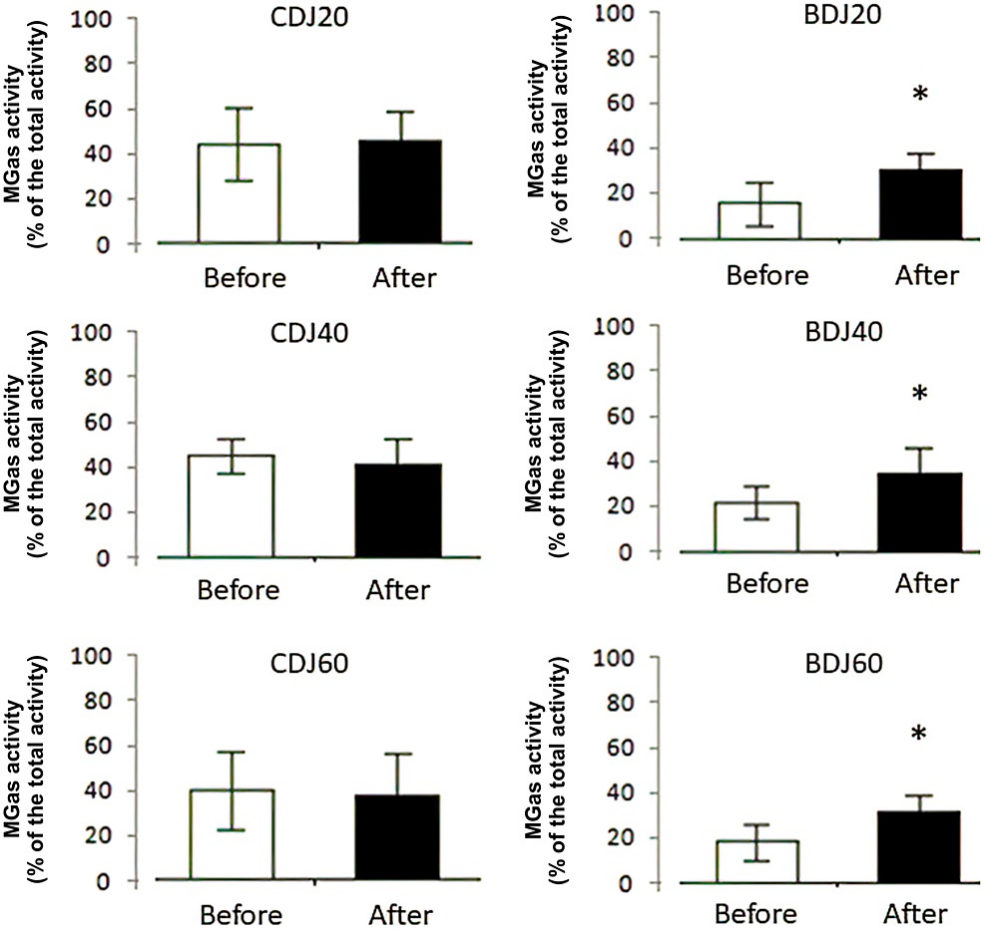
Changes in normalized medial gastrocnemius activity during concentric phases of the drop jumps after inclined hopping intervention * indicates significant differences with the pre-values (p < 0.05). MGas: medial gastrocnemius, CDJ20: counter drop jump from 20 cm, CDJ40: counter drop jump from 40 cm, CDJ60: counter drop jump from 60 cm, BDJ20: bounce drop jump from 20 cm, BDJ40: bounce drop jump from 40 cm, BDJ60: bounce drop jump from 60 cm

The analysis (RM-ANOVA) revealed a statistically significant interaction between group and time for the normalized MGas activity during the concentric phase of BDJ20 (F_(2,27)_ = 11.33, p < 0.001), BDJ40 (F_(2,27)_ = 7.59, p < 0.05), and BDJ60 (F_(2,27)_ = 15.12, p < 0.05), while no significant interaction was found during the eccentric phase. Post hoc analysis indicated that MGas activity during the concentric phase was significantly increased only in the IH group (Figure 1), while no significant effect was found for the PH and EMS groups. The analysis (RM-ANOVA) revealed no statistically significant interaction between group and time for TA, RF, and ST muscle activity in both eccentric and concentric phases in CBJs and BDJs.

**Figure 2:**
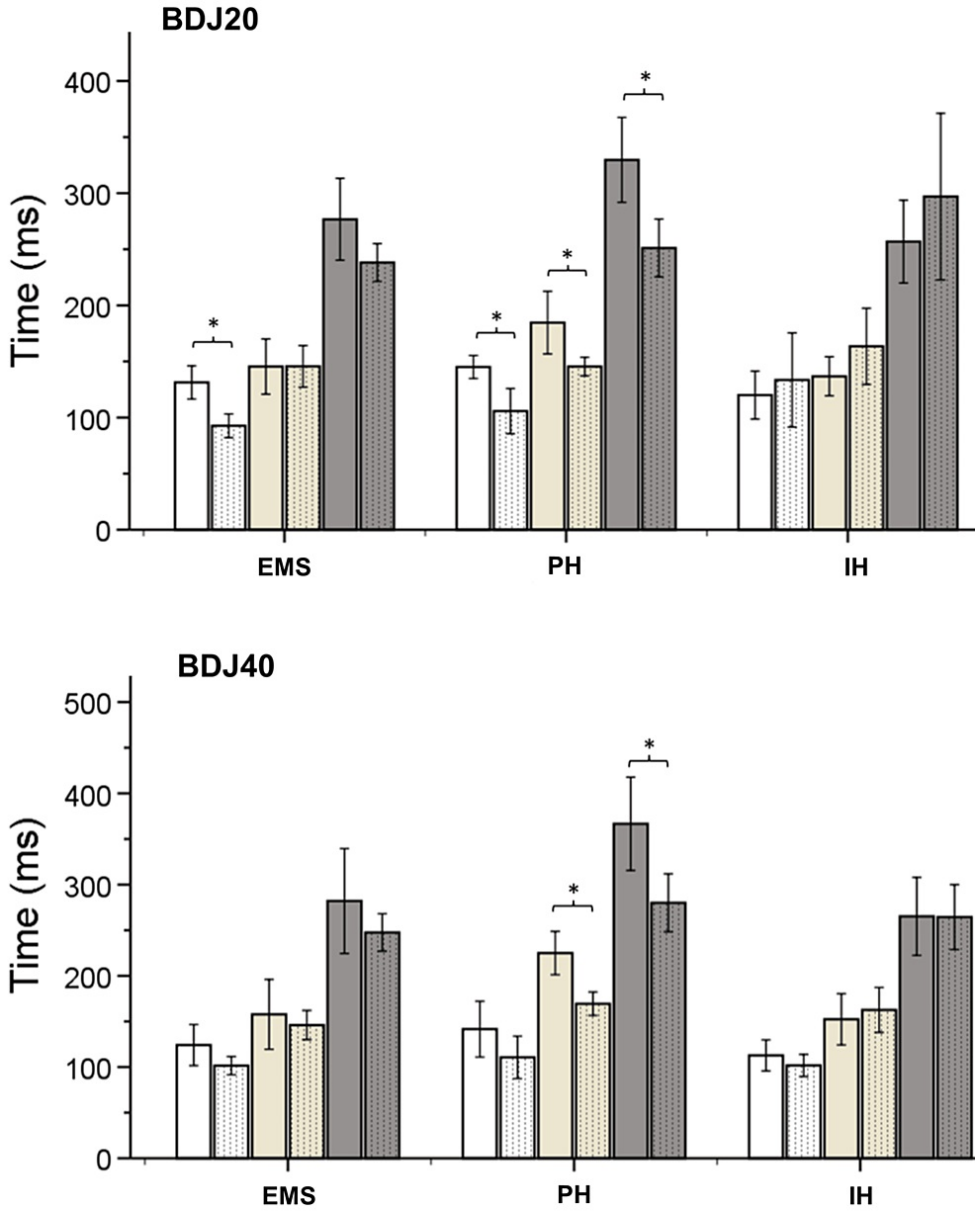
Contact time of eccentric (white shades) and concentric phase (yellow shades), and the total time (gray shades) of bounce drop jump from 20 cm (upper) and bounce drop jump from 40 cm (lower) at the three training groups The solid colors represent the pre-training values, whereas the dotted colors the after-intervention values. * indicates significant differences from the pre-training values (p < 0.05). BDJ20: bounce drop jump from 20 cm, BDJ40: bounce drop jump from 40 cm, EMS: electromyostimulation, PH: plane hopping, IH: incline hopping

The analysis (RM-ANOVA) revealed a statistically significant interaction between group and time for the dependent variables BDJ20 eccentric CT (F(2, 27) = 6.848, p < 0.005), BDJ20 concentric CT (F(2, 27) = 6.552, p < 0.001), BDJ20 total CT (F(2, 27) = 7.838, p < 0.005), BDJ40 concentric CT (F(2, 27) = 7.268, p < 0.005), and BDJ40 total CT (F(2, 27) = 3.596, p < 0.05). Post hoc analysis indicated that after the intervention, CT decreased significantly in BDJ20 during the eccentric phase in the EMS group (p < 0.002) (Figure 2). Additionally, significant group differences were observed at the PH that CT showed a significant decrease after the intervention in BDJ20 during eccentric (p < 0.005), concentric (p = 0.005), and total (p= 0.001), and in BDJ40 during concentric (p < 0.001) and total contact time (p < 0.001) (Figure 2).

The analysis (RM-ANOVA) revealed a statistically significant interaction between group and time for the eccentric isokinetic torque during 30 (F_(2,27)_ = 7.12, p < 0.01), 60 (F_(2,27)_ = 9.23, p < 0.01), and 120°^.^sec^-1^ (F_(2,54)_ = 7.40, p < 0.01). Post hoc analysis indicated that eccentric torque was increased in the three angular velocities for all training groups. Additionally, the analysis (RM-ANOVA) revealed a statistically significant interaction between group and time for the concentric isokinetic torque during 30 (F_(2,27)_ = 17.60, p < 0.001), 60 (F_(2,27)_ = 19.23, p < 0.01), and 120°^.^sec^-1^ (F_(2,27)_ = 17.40, p < 0.01). The results of post hoc analysis indicated that concentric torque was increased in the three angular velocities only for the IH group.

## Discussion

The present study examined the effects of ankle's specific training on produced isokinetic torque, drop jumping dynamics, and kinematics. The present findings clearly showed that IH caused improvements in BDJ V_to_, accompanied by a higher MGas activity during the propulsion phase. Despite the increases in drop jumping height, the joint's kinematics did not alter. The PH and EMS groups did not show any changes in the drop jumping dynamics and kinematics after four weeks of training. All training groups increased eccentric isokinetic torque at all angular velocities, whereas concentric isokinetic torque was increased only after IH training. The present findings extend our knowledge of the effect of plantar flexor's specific training on the ankle's torque and jumping performance.

Our findings showed that IH is effective in increasing jumping ability by altering the jumping height during fast drop jumps, which is consistent with previous studies on incline plyometrics [9]. Given the relatively short duration of the training period (four weeks), it is reasonable to assume that neural adaptations contributed to the changes in vertical jumping. Specifically, our findings indicated increased activity in MGas after the intervention period. The activity of MGas increased, while TA activity remained stable, resulting in a decreased CI. These results align with previous reports suggesting increased EMG activity of the agonist muscle (plantar flexors) following plyometric training [20]. Additionally, it was demonstrated that MGas activity was increased, during the positive phase of drop jump, following ankle-specific plyometric training. Both agonist and antagonist muscle activity can influence force production during vertical jumping [21]. Specifically, during the propulsion phase of fast drop jumps, an increase in MGas activity, with stable TA activity, leads to greater force production at the ankle joint. Therefore, the increase in the propulsion phase can be attributed to the greater force production of the plantar flexors.

Maximal isokinetic plantar flexion torque significantly increased after IH, specifically at 120°, 60°, and 30°^.^sec^-1^ in both eccentric and concentric conditions. Thus, our data on isokinetic torque support the rationale that the increases in BDJs can be explained by the higher force capacity of the plantar flexors. The biomechanical analysis further supports this explanation, as our examination of joint kinematics revealed no significant modifications in joint angles or angular velocities after the intervention period. Therefore, the increased force capacity of the plantar flexors during drop jumping is not attributed to changes in the ankle joint range of motion or angular velocity. While the properties of the aponeurosis were not evaluated in this study, a previous study [11] suggested that inclined plyometrics can improve jumping ability resulting in enhanced force production [22-24] and a more efficient stretch-shortening cycle, by increasing aponeurosis compliance. Although joint angles and velocities remained unchanged, the vertical take-off velocity (V_to_) increased after IH training, likely indicating greater elastic energy storage during the propulsion phase of jumping [25-27]. These findings highlight the importance of considering aponeurosis properties and their impact on muscle function and performance in athletic training programs.

Our findings indicated that plane hopping did not have an effect on vertical drop jumping ability. These results are in agreement with a previous study [9] and contribute to our understanding of adaptations in plyometric training. Consistent with previous reports, the exclusive use of plane hopping does not provide sufficient stimulus to increase jumping ability. The altered neuromuscular activation of the plantar flexors and/or dorsiflexors may contribute to changes in plantar flexion force production during drop jumping [21]. However, the activation of agonist and antagonist muscle groups did not change during either the eccentric or concentric phase of drop jumping. Therefore, our findings regarding neuromuscular activation support the limited benefits of using plane hopping alone. Another possible mechanism that could explain modifications in drop jumping ability is joint kinetics. However, our results regarding joint angles and angular velocities showed no significant changes in any of the joints. These findings align with previous reports that plyometric training fails to change kinematic features in vertical jumping [10,12], especially after a short-term intervention. Considering the results regarding neuromuscular activation and kinematics, it appears unlikely that force or work production during drop jumping can be altered, thus limiting the changes in produced power that could be achieved through plane hopping.

An interesting finding was that the plane hopping intervention resulted in increased eccentric torque. During the eccentric phase of plyometric training, individuals are required to actively resist while generating high levels of force. Plyometric training typically affects the neuromuscular system first, leading to improved motor unit recruitment and synchronization [7]. These adaptations can influence the recruitment of fast motor units during force production, resulting in increases in eccentric torque. Fast motor unit recruitment is a determining factor in eccentric torque production, as they are activated during such muscle actions. Despite the increases in eccentric velocities during isokinetic conditions, there were no beneficial effects on jumping performance. During DJs, especially in fast conditions, the musculotendon system generates force to absorb the body mass, store elastic energy, and release it during the subsequent concentric phase. The effective transition between the braking and propulsion phases requires a limited contact time during the initial phase. Our data on contact time revealed significant decreases in contact time during both the eccentric and concentric phases of BDJ from 20 cm. While the decrease in eccentric contact time supports the utilization of elastic energy, a similar decrease in propulsion contact time may negatively affect the force capacity of the plantar flexors according to the force-velocity relationship. Similarly, a reduction in concentric contact time was observed in BDJs from 40 cm, potentially impairing force production from the plantar flexors. Based on these findings, it is reasonable to assume that PH fails to cause any modifications in neuromuscular function, kinetics, and dynamics of drop jumping. Plane hopping may be appropriate and can be used in an introductory cycle of plyometric training or in combination with other exercises.

Regarding the adaptations observed in EMS training, our findings demonstrate that EMS training resulted in improvements in maximal torque during eccentric conditions in the plantar flexors. These findings are consistent with previous studies [16,19] that also reported significant increases in muscle force of the plantar flexors in trained individuals. As mentioned earlier, the adaptations that occur following short-term EMS training are likely to be neural in nature [28]. During EMS training, the stimulus induces motor unit recruitment without being limited by their types [29]. Consequently, even the fast motor units can be activated at lower force levels than maximal. Additionally, contractions during EMS training are characterized by synchronous recruitment of motor units [14]. These neural characteristics during EMS training result in a greater number of activated motor units, including the fast ones with a greater force capacity, which is particularly important for eccentric actions. Therefore, EMS training leads to increased eccentric torque due to a greater overall activation and more synchronous recruitment of motor units, including a higher proportion of fast motor units.

In contrast to the improvements observed in eccentric torque, our results showed that EMS training did not have a significant effect on drop jumping performance. These findings are inconsistent with previous reports that demonstrated improvements in sprinting and vertical jumping following EMS training [19]. For instance, previous findings showed that a 12-week EMS intervention program improved both squat and drop jumping performance [20]. One possible reason for the discrepancies in results could be the training procedure. In both of the aforementioned studies, EMS training was combined with voluntary "sport-specific" exercises. In contrast, our intervention in the EMS group solely consisted of EMS stimulus. The electrical current was applied isometrically at -15° of the ankle joint, providing a general stimulus to the plantar flexors. Despite the increases in plantar flexor torque capacity, EMS training may have impaired jumping coordination, resulting in inefficient use of the generated force. Our analysis of drop jumping revealed no significant modifications in dynamics and kinetics parameters following EMS intervention. These findings support the notion that EMS training should be used as a supplemental training method to increase muscle strength and should be applied in conjunction with sport-specific exercises to improve specific performance.

Limitations

The short duration of the intervention program should be considered a limitation of this study. The four-week training program might not be an efficient period to affect the technique of drop jumping and the joint’s coordination. Thus, a longer intervention period might be more efficient to change the aforementioned parameters. Despite the small sample of the three intervention groups, the power analysis revealed that a minimum sample size of eight participants per group would be needed to achieve the desired power. In addition, this study did not include a control group as in previous studies. However, the purpose of the study was to compare the adaptations caused by the three ankle-specific protocols in drop jumping ability. Plane hopping acts as a control group, since it is the most commonly used plyometric exercise for plantar flexors. Moreover, as our sample consisted of only male participants, the extrapolation of the present results for female subjects requires caution. Further research should be conducted to evaluate whether similar adaptations also occur in female youth or adults.

## Conclusions

In conclusion, the present study highlights the importance of specialized training regimens for enhancing drop jumping ability. Incline hopping showed significant improvements in jumping performance, likely due to neural adaptations and increased force production by the plantar flexors. The compliance of the MGas aponeurosis may play a role in these improvements by enabling greater force production and efficient energy storage during the stretch-shortening cycle. In contrast, plane hopping alone did not lead to significant improvements in jumping ability, suggesting its limited benefits as a stand-alone exercise. EMS training resulted in increased eccentric torque in the plantar flexors, but its impact on drop jumping performance was not significant. The study suggests that EMS training might be beneficial when combined with sport-specific exercises to enhance overall athletic performance. However, further research with longer intervention periods and varied training protocols is needed to fully understand the effects of specific training on jumping biomechanics. Overall, the findings provide valuable insights into the role of different training regimens in improving drop jumping ability and highlight the importance of combining specialized exercises to optimize athletic performance.
